# Merkel cell carcinoma in Singapore

**DOI:** 10.1016/j.jdin.2025.12.009

**Published:** 2025-12-31

**Authors:** Trang T.T. Chu, Zhi Yi Alicia Seet, Ellie C.E. Choi, Peter K.C. Goon

**Affiliations:** a Department of Medicine, Yong Loo Lin School of Medicine, National University of Singapore, Singapore; b Division of Dermatology, Department of Medicine, National University Hospital, Singapore; c Department of Otolaryngology, Yong Loo Lin School of Medicine, National University of Singapore, Singapore

**Keywords:** Asia, ASIs, epidemiology, Merkel cell carcinoma, Singapore, skin of color

*To the Editor:* Merkel cell carcinoma (MCC) is a rare but highly aggressive, life-threatening skin cancer^1^^,^^2^ with poor prognosis and high recurrence. Patients are predominantly fair-skinned, aged >75 years, with the cancer occurring on ultraviolet-exposed body sites.^1^^,^^3^ MCC incidence is increasing worldwide, with a >95% rise in the United States from 2000-2013 (0.7 expected cases per 100,000 person-years),^2^ and a threefold increase in the United Kingdom from 2004-2013.^1^ Increasing crude incidence rates from Asia have also emerged,^3^^,^^4^ but none report age-standardized incidence rates (ASIs) allowing for direct comparison.

This study aimed to (1) analyze MCC ASIs in Singapore relative to steep increases in the West and (2) estimate case numbers for 2030 onwards. Singapore has comparable GDP and living standards to the West, but with strong year-round equatorial UV exposure and a predominantly Asian population (Fitzpatrick skin types 3-6).

Deidentified data from the Singapore National Registry of Diseases Office (population-based) were retrieved (01/01/1968-31/12/2022). MCC cases were retrieved with International Classification of Diseases (ICD) categories ICD, Ninth Revision (1968-1992), ICD for Oncology, Second Edition (1993-2002), ICD for Oncology, Third Edition (2003 onward), and histology code (8247/3). Singapore’s first MCC case in 1997 determined the study period of 1997-2021.

32 MCC cases (male = 16, female = 16) were identified in Singapore from 1997-2021 (Fig 1). Except for 1 patient aged 30-34 years, age at diagnosis was 63.0-95.8 (median = 82.1) years, with 62.5% (20) of patients aged ≥80 years. MCC incidence is increasing—12 cases were diagnosed from 1997-2007 and 20 over the same duration from 2011-2021 (Table I). Median survival time postdiagnosis was 1.6 years only. ASI was 0.035 expected cases per 100,000 person-years referenced to the standard world population. Disease incidence was highest in patients aged 80-84 years (ASI = 0.07**4**) and ≥90 years (ASI = 0.058). Predicted future incidence was extrapolated via simple linear regression (Fig 1), with a projected increase of 8 new cases per 5 years by 2030 and 10 new cases per 5 years by 2040.

**Fig 1:**
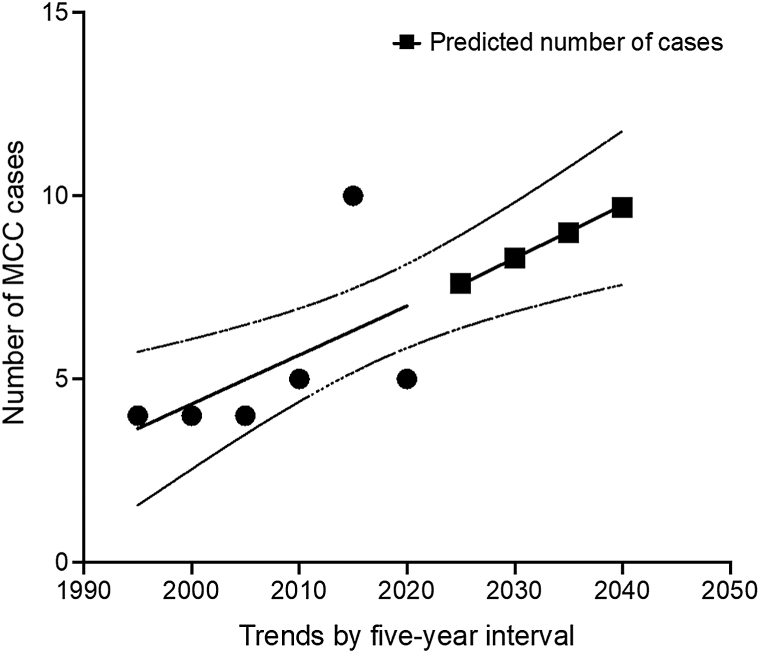
Number of cases of MCC in Singapore, 1997-2021 (in 5-year blocks). *MCC*, Merkel cell carcinoma.

**Table I:** ASIs for Merkel cell carcinoma in Singapore

Age group, *i*	No. of cases, d_*i*_	Person-years at risk, y_*i*_ (1997-2021)	Age-specific incidence (per 100,000 person-years) 100000∗ (d_*i*_/y_*i*_)	WHO World Standard population, w_*i*_	Expected cases in standard population, d_*i*_w_*i*_/y_*i*_
0-4	0	5,001,272	0	88,569	0.000
5-9	0	5,607,829	0	86,870	0.000
10-14	0	5,794,372	0	85,970	0.000
15-19	0	5,828,362	0	84,670	0.000
20-24	0	5,923,551	0	82,171	0.000
25-29	0	6,682,308	0	79,272	0.000
30-34	1	7,329,875	0	76,073	0.010
35-39	0	7,716,109	0	71,475	0.000
40-44	0	7,766,387	0	65,877	0.000
45-49	0	7,452,754	0	60,379	0.000
50-54	0	6,705,986	0	53,681	0.000
55-59	0	5,681,214	0	45,484	0.000
60-64	1	4,532,954	0.022	37,187	0.008
65-69	2	3,397,815	0.059	29,590	0.017
70-74	5	2,403,571	0.208	22,092	0.046
75-79	3	1,589,453	0.189	15,195	0.029
80-84	8	988,660	0.809	9097	0.074
85-89	3	545,775	0.550	4398	0.024
>= 90	9	300,508	2.995	1950	0.058
Total	**32**	91,197,049	0.035	**100,000**	0.035

*ASIs*, Age-standardized incidence rates; *WHO*, World Health Organization.

∗ For purposes of comparison, the WHO Standard age group ≥90 is an aggregate of the age groups 90-94, 95-99, and 100+.

MCC incidence in Singapore remains relatively rare, with a significantly lower ASI (0.035) than that of well-established cohorts in the United Kingdom (0.65),^3^ the United States (0.7),^2^ and Australia (1.6).^3^ The rarity of MCC in Asian populations means that epidemiology data are sparse and may not align with trends observed in the West.

The observed 1:1 sex ratio differs from the male preponderance previously described in largely Eurocentric cohorts^1^, ^2^, ^3^ and conversely the female predominance in smaller Asian cohorts. Further research is required to ascertain if this is a characteristic of the Singaporean population or simply an artefact.

87.5% (28) of cases were of Chinese ethnicity, exceeding that of Singapore’s 74.3% Chinese population.^5^ This may be attributed to fairer skin as an established MCC risk factor, with the highest incidence rates from Western cohorts comprising more than 90% White patients.^3^ Merkel cell polyomavirus infection/integration into MCC is now an established risk factor in the West and will be a vital area of future research in Singapore.

This study presents analysis of MCC ASIs in Singapore over 25 years and estimates of future incidence rates. While the overall burden of MCC in Singapore remains low, increasing incidence rates and the lethality of the cancer pose a growing concern.

## Conflicts of interest

None disclosed.
